# Pharmacokinetics and Bioavailability Study of Monocrotaline in Mouse Blood by Ultra-Performance Liquid Chromatography-Tandem Mass Spectrometry

**DOI:** 10.1155/2018/1578643

**Published:** 2018-08-13

**Authors:** Lianguo Chen, Bin Zhang, Jinlai Liu, Zhehua Fan, Ziwei Weng, Peiwu Geng, Xianqin Wang, Guanyang Lin

**Affiliations:** ^1^The Third Clinical Institute Affiliated with Wenzhou Medical University & Wenzhou People's Hospital, Wenzhou 325000, China; ^2^Analytical and Testing Center, School of Pharmaceutical Sciences, Wenzhou Medical University, Wenzhou 325035, China; ^3^Laboratory of Clinical Pharmacy, The People's Hospital of Lishui, Lishui 323000, China; ^4^The First Affiliated Hospital of Wenzhou Medical University, Wenzhou 325000, China

## Abstract

**Background and Aims:**

The present study aimed to develop a simple and sensitive method for quantitative determination of monocrotaline (MCT) in mouse blood employing ultra-performance liquid chromatography-electrospray ionization tandem mass spectrometry (UPLC-ESI/MS/MS) using rhynchophylline as an internal standard.

**Methods:**

Proteins present in the blood samples were precipitated using acetonitrile. MCT was separated using a 1.7-*μ*m ethylene bridged hybrid (BEH) C18 column (2.1 mm × 50 mm) with a gradient elution program and a constant flow rate of 0.4 mL/min. The LC mobile phase consisted of 10 mmol/L ammonium acetate (containing 0.1% formic acid) and acetonitrile. The total elution time was 4.0 min. The analytes were detected on a UPLC-ESI mass spectrometer in multiple reaction monitoring (MRM) mode and quantified.

**Results:**

The new method for the determination of MCT has a satisfactory linear detection range of 1-2000 ng/mL and excellent linearity (*r* = 0.9971). The lower limit of quantification (LLOQ) of MCT is 1.0 ng/mL. Intra- and interassay precisions of MCT were ≤13% with an accuracy from 96.2% to 106.6%. The average recovery of the new method was >75.0%, and matrix effects were between 89.0% and 94.3%. Based on the pharmacokinetics data, the bioavailability of MCT in mice was 88.3% after oral administration.

**Conclusions:**

The results suggest that the newly standardized method for quantitative determination of MCT in whole blood is fast, reliable, specific, sensitive, and suitable for pharmacokinetic studies of MCT after intravenous or intragastric administration.

## 1. Introduction

Monocrotaline (crotaline, MCT), a pyrrolizidine alkaloid (PA) isolated from* Crotalaria* species, induces toxicity in many tissues and causes extreme hepatic necrosis, pulmonary hypertension, and severe kidney damage [1]. MCT is considered not suitable to continuously use as a drug and is mainly used to induce pulmonary diseases in mice [2, 3]. In 1991, Mattocks et al. [4] first reported that 7-glutathionyl-dehydroretronecine (GS-DHR) given to rats was able to mimic the cardiopulmonary toxicity of MCT. In recent years, it was found that MCT exhibits dose-dependent cytotoxicity with potent antineoplastic activity [5, 6]. Thus, lower doses of MCT may be a potent anticancer or cytotoxic agent in combination with other protective agents, which remains to be confirmed by further* in vivo* studies.

It is well known that pharmacokinetic studies play a pivotal role in drug development, as they assist in predicting a variety of efficacy- and toxicity-related events. To better understand how the toxicity and the pharmacological activity of MCT* in vivo* change with the blood concentration, a rapid, simple, and effective analytical method is necessary. Up to the present moment, only a few bioanalytical methods have been published for the detection of MCT in biological fluids. Estep et al. [7] reported the results of urinary and biliary excretion, and plasma kinetics of [14C]MCT by high-performance liquid chromatography (HPLC) in 1991, but the HPLC method was less effective because of the long detection time for MCT (approximately 40 min). Glowaz and Wang et al. [8, 9] also briefly mentioned that HPLC was used for detection of a reactive pyrrole in the hepatic metabolism of MCT, metabolites formed from the metabolism of MCT, and the chromatographic peak of MCT in their research eluted at 12.4 min and 28.9 min, respectively. Unfortunately, they did not elaborate on the sample pretreatment and HPLC conditions. It is undeniable that HPLC is inexpensive and commonly used, but the weakness is also apparent: low selectivity, low sensitivity, and long detection time. Thus, Yao et al. [1] chose the HPLC/MS/MS method for detecting MCT and its metabolites in the plasma, bile, and tissues of mice for many advantages: simplified sample preparation, high sensitivity, selectivity, and reliability. Although the published studies described above provide useful information, little data are available for MCT determination. The UPLC and UPLC-MS/MS techniques have recently attracted additional interest along with the development of analysis techniques [10, 11]. Compared with HPLC-MS/MS, the advantages of UPLC-MS/MS include fast analysis, high throughput, and less solvent required [12–16].

As far as we know, there are no published data that demonstrate the validation of a sensitive assay utilizing UPLC-MS/MS for the determination of MCT in whole blood. Therefore, we standardized and validated a new and more convenient UPLC/MS/MS method in this study for the determination of the concentration of MCT, utilizing the serial blood sampling method and performing it in 4 min. After adding rhynchophylline (IS), protein precipitation was used to extract analytes. This method had been successfully applied to the pharmacokinetic study of MCT in mice after sublingual intravenous and gavage administration.

## 2. Materials and Methods

### 2.1. Drugs and Reagents

**Figure 1:**
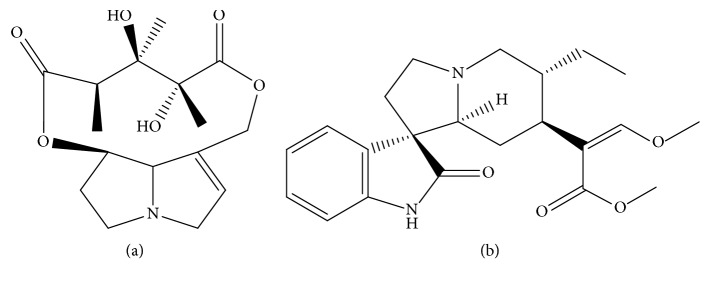
Chemical structures of (a) monocrotaline and (b) rhynchophylline.

Monocrotaline (purity > 98%, Figure 1(a)) and rhynchophylline (IS, purity > 98%, Figure 1(b)) were purchased from Chengdu Mansite Pharmaceutical Co. Ltd. (Chengdu, China). HPLC-grade formic acid and organic solvents (acetonitrile and methanol) were purchased from Tedia (Ohio, USA) and Merck (Darmstadt, Germany), respectively. Purified water was obtained with a Milli-Q purification system (Millipore, Bedford, MA, USA).

### 2.2. Animals

Male ICR mice (20-22 g; n=12) were obtained from the Laboratory Animal Center of Wenzhou Medical University (Wenzhou, China). The study protocol was approved by the Animal Care Committee of Wenzhou Medical University. Mice received standard food and water* ad libitum* in a temperature-controlled room (25°C) with 12-h on and 12-h off light cycle before experiments.

### 2.3. UPLC-MS/MS Conditions

A UPLC-MS/MS system with an ACQUITY I-Class UPLC and a XEVO TQ-S micro triple quadrupole mass spectrometer (Waters Corp., Milford, MA, USA) was used for analysis. The output signal monitoring and processing were performed by MassLynx 4.1 software (Waters Corp.).

Symmetric peak performance and satisfactory retention of the analytes were accomplished on a 1.7 *μ*m UPLC BEH C18 (2.1 mm × 50 mm) column. Acetonitrile (solvent A) and 10 mmol/L ammonium acetate (solvent B, containing 0.1% formic acid) were selected as the mobile phase. The gradient program consisted of the following: 10% A (0-0.2 min); changing to 80% A (0.2-1.5 min); 80% A (1.5-2.0 min); changing to 10% A (2.0-2.5 min); and 10% A (2.5-4.0 min). The flow rate was set at 0.4 mL/min, the column temperature was 40°C, and the total elution time was 4.0 min.

Ionization was achieved by using electrospray in the positive ion mode (ESI+). The MRM mode was applied to monitor MCT ions at* m*/*z* 326.2→120.8. The cone voltage for MCT was set to 78 V and the collision voltage to 30 V. For IS, the MRM transition was* m*/*z* 385.2→160.0 with a cone voltage of 36 V and a collision voltage of 34 V. The capillary voltage was set to 2.1 kV for both MCT and IS. The desolvation gas (nitrogen) was set at 800 L/h with the cone gas at 50 L/h. The temperature of the ion source and desolvent was 150°C and 400°C, respectively.

### 2.4. Standard Solution Samples

Standard stock solutions of MCT and IS were prepared in methanol at 1 mg/mL. Then, these stock solutions of MCT were diluted with methanol to obtain fresh standard working solutions at several concentration levels. The standard working solution of IS was diluted with acetonitrile to the concentration of 20 ng/mL. All solutions were stored at 4°C before analysis.

### 2.5. Calibration Standards (CS) and Quality Control (QC) Samples

CS and QC samples were prepared by diluting corresponding standard working solutions with the blank blood of mice. The concentrations of the calibration standard were 1, 5, 10, 20, 50, 100, 200, 1000, and 2000 ng/mL. Three concentrations of QC samples representing the entire range of the standard curve were prepared at 3, 180, and 1800 ng/mL: one within 3X LLOQ (low-level QC sample), one near the center (mid-level QC sample), and one near the upper boundary of the standard curve (high-level QC sample). The CS and QC samples were maintained at −20°C until processing.

### 2.6. Sample Preparation

Frozen blood samples (20 *μ*L) in 1.5-mL test tubes were brought to room temperature before adding 100 *μ*L acetonitrile (containing 20 ng/mL of IS). Then, these tubes were mixed on a vortexer for 60 seconds before 10 min of centrifugation (13,000 rpm, 4°C). The supernatant (approximately 80 *μ*L) was collected into a new micro-insert (clear glass, cone-shaped with a plastic stent), and then 2 *μ*L of the supernatant was injected into the UPLC-MS/MS system.

### 2.7. Method Validation

The method was validated for selectivity, linearity, accuracy, precision, recovery, stability, and matrix effects of samples according to the “Guideline on Bioanalytical Method Validation” recommended by the US Food and Drug Administration (FDA) in 2013 [17].

#### 2.7.1. Selectivity

The selectivity was evaluated by analyzing blank mouse blood, blank blood spiked with MCT and IS, and a mouse blood sample after dosing. The method was established without interference from endogenous peaks existing at the peak region of MCT and IS in the blank blood.

#### 2.7.2. Linearity

Calibration curves were generated by analyzing different concentrations of calibration samples on three consecutive days. The linear regressions of the peak area ratios (y) of each MCT to the corresponding IS versus the nominal concentration (x) of MCT were fitted over the range of 1-2000 ng/mL. Linearity was evaluated at 9 levels covering the concentration range of 1-2000 ng/mL.

#### 2.7.3. Precision and Accuracy

Evaluating the intraday and interday accuracy and the precision across the quantitation range during method standardization is essential and involves analyzing QC samples at multiple concentrations across the assay range. Method validation experiments for estimating accuracy and precision should include a minimum of three levels (3, 180, 1800 ng/mL for MCT) and six independent runs conducted on the same day and three consecutive days. The precision and the accuracy were expressed by the relative standard deviation (RSD) and relative error (RE), respectively, which should be within the limits of ±15% at all concentrations.

#### 2.7.4. Recovery and Matrix Effect

The recovery of MCT was calculated by comparison of the peak area responses of the QC samples (n=6) that were added before extraction and the IS that was subsequently added at three concentrations (3, 180, 1800 ng/mL), with those obtained when both the corresponding MCT and IS were added after the extraction step.

Before evaluating the matrix effect, the stock solutions of MCT were diluted with the extracted blank blood to get new working solutions at three levels (3, 180, 1800 ng/mL). Then, matrix effects were tested by comparison of the peak areas of these new working solutions with those of the corresponding standard solutions diluted with acetonitrile: 0.1% formic acid (1:1, v/v) at equivalent concentration, and this peak area ratio was defined as the matrix effect.

#### 2.7.5. Stability

The stability of MCT in mouse blood was evaluated by analyzing blood samples containing QC samples at low, medium, and high concentration levels (n=3 for each concentration level). The MCT stability was tested under the following conditions: (a) storage for 12 h at room temperature in an autosampler; (b) storage for 30 days at −20°C; and (c) three complete freeze-thaw cycles (−20°C to room temperature).

### 2.8. Pharmacokinetic Study

MCT was dissolved in 0.01% HCl solution for administration to mice and freshly prepared before the experiment. The mice were divided into two groups (group A and group B,* n* = 6 for each group): the mice in group A were treated with a single sublingual intravenous injection of MCT at 3 mg/kg after 12 h fasting, while the others in group B were administered an oral dose of MCT at 15 mg/kg. Blood samples (20 *μ*L) were collected in 1.5-mL tubes containing heparin by tail tip bleeding at 0 (prior to dosing), 0.083, 0.5, 1, 1.5, 2, 3, 4, 8, 12, and 24 h after dosing of MCT, and stored directly at −20°C until analysis. Blood samples were processed for UPLC-MS/MS analysis according to the method described in Sample Preparation. DAS software (version 2.0, China Pharmaceutical University, China) was used to calculate the main kinetic parameters, such as area under the concentration-time curve (AUC), the half-life (t_1/2_), peak blood concentrations (C_max_), clearance (CL), mean resident time (MRT), and volume of distribution (V). In addition, the bioavailability of MCT was examined in our study for the first time and was calculated as absolute bioavailability (%) = 100×AUC_po_ · D_iv_ / (AUC_iv_ ·D_po_), where AUC_iv_ and AUC_po_ are the AUC of the drug from (0 - ∞) after intravenous and oral administration and D_iv_ and D_po_ are a single dosage of MCT for intravenous and oral administration, respectively.

## 3. Results

### 3.1. Method Validation

#### 3.1.1. Selectivity

Figure 3 presents the ion chromatogram of a blank extract, a blank extract with MCT and IS, and an authentic sample spiked with IS. The peaks of MCT and IS appeared at 0.46 and 1.71 min, respectively. The separation of MCT and IS was satisfactory. No interfering peaks were found at or near the retention times of MCT and IS. There was increased sensitivity and selectivity when UPLC-MS/MS was used for the quantitative determination of MCT utilizing 20 *μ*L mouse blood as compared to the traditional HPLC method. The total runtime was 4.0 min per sample (including equilibration time), which is important for large batches of samples and faster than the LC-MS method (more than 10 min) developed by Yao et al. [1].

#### 3.1.2. Calibration Curve

A linear relationship was observed in the calibration curves over the concentration range of 1-2000 ng/mL for MCT in mouse blood. The regression equation is expressed as y = 0.000368x-0.000344, r=0.9971, where y represents the peak ratio of the MCT peak area to IS and x represents the concentration of MCT in mouse blood. The LLOQ was 1 ng/mL with a signal-to-noise (S/N) ratio of 9 for the determination of MCT in mouse blood, which will contribute to the assay of lower concentrations of MCT at the last time point for sample collection.

#### 3.1.3. Accuracy and Precision

As shown in Table 1, the results of intra- and interday precision assessed by the relative standard deviation (RSD) were no more than 12% and 13%, respectively. The accuracy was in the range of 96.2-106.6% at each QC level. All of the recoveries were above 75.0%, and matrix effects were between 89.0% and 94.3%. These data suggest that both precision and accuracy are within the acceptable range, and the UPLC-MS/MS method established is suitable for the pharmacokinetic study of MCT.

#### 3.1.4. Recovery and Matrix Effects

As can be seen from Table 1, the recovery for the method was in the range 75.0%-81.9% with matrix effect within the range of 89.0–94.3%. The results indicate reasonable recoveries with a negligible matrix effect for this method.

#### 3.1.5. Stability

The stability studies for MCT in the blood of mice were performed for each concentration (3, 180, 1800 ng/mL) under the different storage conditions mentioned above (n=3). As can be seen from Table 2, the RSDs were ≤ 14% in all stability tests for MCT, which indicated reliable stability behavior for MCT under the different storage conditions.

### 3.2. Pharmacokinetics

The mean blood concentration-time curves for MCT after intravenous and intragastric administration are shown in Figures 4 and 5, respectively. The main pharmacokinetic parameters after intragastric (15 mg/kg) and sublingual intravenous (3 mg/kg) administration based on noncompartment model analysis are presented in Table 3. The high bioavailability of MCT (88.3% in this study) as well as short t_max_ (0.5 h) after oral administration indicated that MCT was quickly absorbed and less affected by the liver (or intestinal) first pass effect in animals. Thus, it would be expected that MCT could be developed for oral administration in a solid dosage form and used for treatment in the future.

## 4. Discussion

It is known that the pharmacokinetic profile and toxicity of some drugs are variable in different species [18, 19]. The mouse was chosen as the animal model in this study because it is one of the most common species for evaluating drug preclinical efficacy [20, 21], toxicology [22], biodistribution, and pharmacokinetics [23–25].

Terminal blood sampling has been widely adopted in the pharmacokinetic evaluation of mice, but it is inappropriate for protection of animals because it requires large numbers of animals at high cost and labor. Furthermore, individual animal differences and administration errors may lead to inaccuracy of the pharmacokinetic profile [26]. A serial blood sampling method, by contrast, can reduce the number of animals needed, labor, and cost [27]. In addition, the standard deviation of pharmacokinetic parameters can be calculated based on individual drug concentrations by the serial blood sampling method, while it cannot be calculated based on mean drug concentrations by the terminal blood sampling method. Thus, the data obtained by the serial blood sampling method are more reliable than those obtained by the terminal blood sampling method.

Small blood volume requirements for mouse pharmacokinetic evaluations support serial blood sampling and enable an entire pharmacokinetics profile to be obtained from a single mouse [20, 27]. In reality, the total blood volume of rodents is approximately 7% of their body weight [28]. Thus, the volume of a blood sample that can be collected from a mouse (approximately 20 g) is limited. Typically, blood samples (approximately 20-30 *μ*L) for 6-7 time points are withdrawn from an individual mouse, and six separate mice are used for sample collection and analysis at each time point [20]. Unfortunately, the volume of blood collected in Yao's study was not mentioned and no references were provided. Taking these factors into consideration and on the basis of previous work, we established an improved method for serially sampling the blood (11 total time points in 24 h) from one mouse with only one incision of the lateral tail vein at the first sampling time and sufficient warming of the tail at subsequent sampling times, which imparts low stress to mice and improves the quality of the pharmacokinetic study. Sufficient warming of the tail is critical for the rapid and multiple collections of blood samples from a mouse. The volume of blood sampled for each time point was only 20 *μ*L, and the total blood volume sampled from one mouse was approximately 10% of the total circulating blood volume.

Yao et al. briefly mentioned that HPLC/MS/MS was used for the detection of MCT in the plasma of mice. Their study, however, was focused on the relationship of the hepatic cytochrome P450s and monocrotaline-induced renal toxicity in mice. Therefore, they did not elaborate on the analytical procedure such as selectivity, accuracy, stability, quantification range, linearity, and matrix effect. The current method is an improvement to the published data by Yao et al. because the featured technique is more sensitive with a higher throughput and uses less solvent and time.

**Figure 2:**
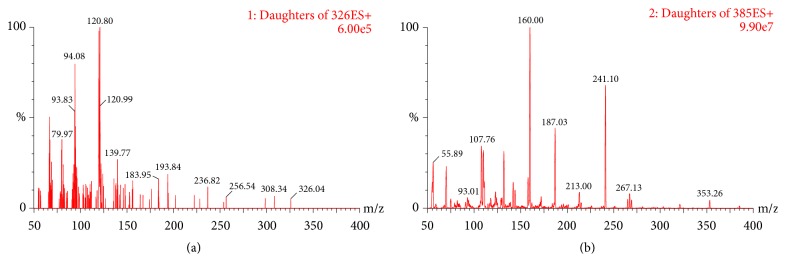
The product ion spectrum of (a) MCT and (b) IS.

**Figure 3:**
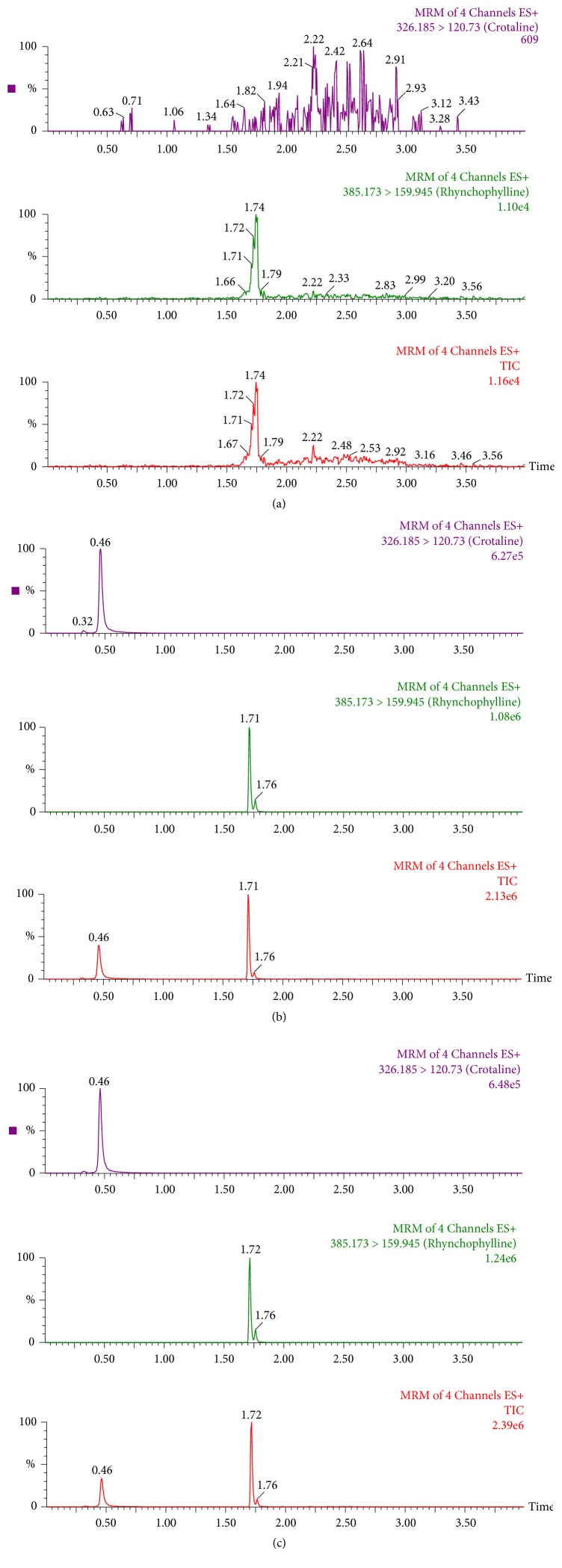
The MS/MS chromatograms of MCT and IS. (a) A blank extract, (b) a blank extract with MCT and IS, and (c) an authentic sample spiked with IS at 0.5 h after intravenous administration.

**Figure 4:**
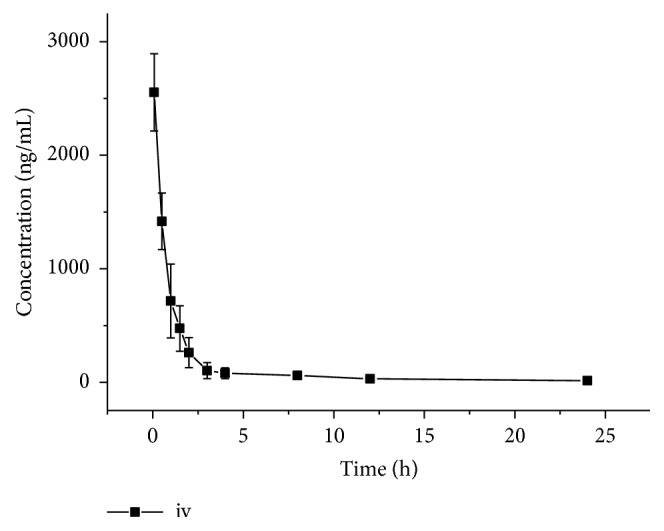
Mean blood concentration of MCT after sublingual intravenous administration at the dose of 3 mg/kg in six mice.

**Figure 5:**
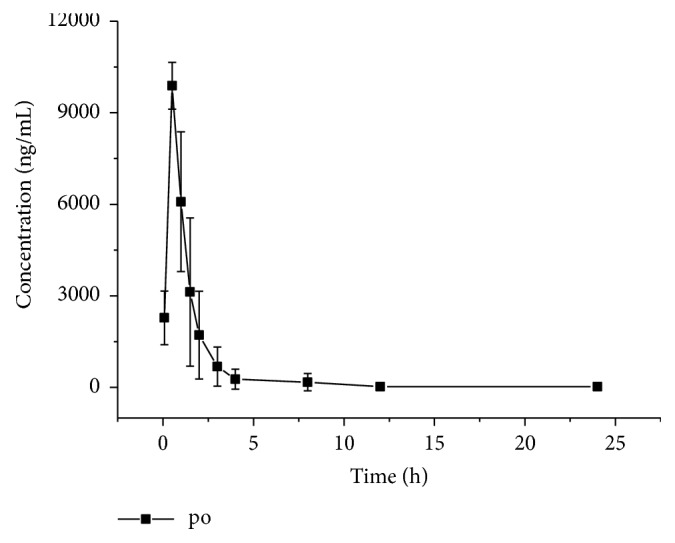
Mean blood concentration of MCT after gavage of 15 mg/kg in six mice.

To optimize the MS conditions, positive and negative ion mode selection was often tested in the methodology. Ultimately, we chose the positive ESI mode for the detection because of the stronger and more stable responses of the analytes as compared to the negative ion mode. According to the optimized results for mass spectrometric conditions, we can see that the daughter ions* m*/*z* 120.8 and* m*/*z* 160.0 were the strongest and the most stable among abundant fragment ions produced by MCT and IS, respectively, which is presented in Figure 2. Thus, we selected* m*/*z* 326.2→120.8 and* m*/*z* 385.2→160.0 for MCT and IS, respectively.

Analysis of MCT with reversed phase UPLC-MS/MS was accomplished with the use of acidic mobile phases, which is more suitable for ion formation of analytes in the electrospray ionization (ESI) source [29, 30]. According to our original work and current conditions, several reversed phase columns were tested (Acquity BEH C18, Ultimate XB C18, and Hanbon Dubhe C18). We chose the Acquity BEH C18 column because of the satisfactory separation and sharper peaks. To avoid endogenous compounds appearing at the same retention times for MCT or IS, a suitable mobile phase was needed. Different acidic mobile phase compositions were tested on an Acquity BEH C18 column to obtain a perfect separation and more symmetrical peak shape, such as acetonitrile-0.1% formic acid, acetonitrile-10 mmol/L ammonium acetate (containing 0.1% formic acid), methanol-0.1% formic acid, and methanol-10 mmol/L ammonium acetate (containing 0.1% formic acid). Acetonitrile-10 mmol/L ammonium acetate (containing 0.1% formic acid) was chosen in this study for the most satisfactory resolution, peak shape, and retention time. Beyond that, gradient elution is more optimal than isocratic elution for sharper peaks and less analysis time.

Two main blood sample processing methods were used before detection: direct analysis of the blood; a sample in which the composition of the blood had been simplified by removing, for example, most of the endogenous substances and proteins. If there is a need to avoid interference or decompose drug-protein complexes, simplified samples are prepared from the blood, most frequently by extraction or by precipitation with the appropriate precipitation agents. Liquid-liquid extraction (LLE) has the advantages of a high extraction rate and low limit of quantification [30]. Yao et al. [1] reported that liquid-liquid extraction with n-butanol was used with MCT. The main disadvantage of extraction is the lengthy sample preparation due to evaporation of the extraction solvent, which results in a method that is time consuming, complicated, and expensive. Moreover, it is difficult to obtain sufficient plasma after centrifuging for liquid-liquid extraction at each point by tail vein transactional bleeding. Thus, Yao et al. selected just 8 total time points for calculating the pharmacokinetic parameters. A one-step protein precipitation procedure for whole blood was chosen in our study following the example of previous studies [31, 32]. The supernatant obtained from the blood after precipitation and centrifugation was directly injected into the column, which significantly simplified the sample preparation and offered a high throughput assay. The precipitation method is more convenient, but only if the level of drugs in the blood is sufficiently high for detection. The LLOQ for MCT (1 ng/mL) in our study is much lower than that (5 ng/mL) achieved by Yao et al., which ensures that the level of MCT in the supernatant obtained from the blood at the last time point after protein precipitation and centrifugation is sufficiently high to be detected by UPLC-MS/MS. Thus, this assay is a modified version of a mass spectrometry assay used to determine MCT in plasma by Yao et al. and Li et al. [1, 31].

The following precipitating agents and their mixtures in different combinations and ratios were tested: methanol, acetonitrile, and acetonitrile–methanol. During the precipitation processes, the volumes of the respective precipitation agents were always the same. The results indicated that the greatest recoveries of the analytes were achieved when acetonitrile was used as the precipitating reagent. Considering that blood samples are more complex than plasma, the 20-*μ*L blood sample was mixed with 5 volumes of acetonitrile, which provided higher recoveries, less matrix effect, and also sufficient supernatant volume for analysis requiring multiple injections.

## 5. Conclusion

A simple, sensitive, and robust method using UPLC/MS/MS for the quantitative measurement of MCT in mouse blood was standardized and validated. The method offers sample extraction from only 20 *μ*L of whole blood using a simple protein precipitation procedure and was successfully applied to the pharmacokinetic investigations of MCT in mice while also meeting the requirement of high sample throughput in bioanalysis. The oral bioavailability of MCT in mice was 88.3%, which indicates that MCT is easily absorbed into the blood circulatory system through the gastrointestinal tract.

## Figures and Tables

**Table 1:** The precision, accuracy, recovery, and matrix effect of MCT in mouse blood (n = 6).

Concentration (ng/mL)	Precision (RSD%)		Accuracy (%)		Found (ng/mL)		Matrix effect (%)	Recovery (%)
	Intra-day	Inter-day	Intra-day	Inter-day	Intra-day	Inter-day		
3	9.6	12.6	106.6	96.6	3.2±0.3	2.9 ±0.4	89.0±7.6	81.9 ±6.4
180	11.1	12.0	99.4	104.4	178.9±19.9	187.9 ±22.6	94.3±4.5	77.2±3.6
1800	7.3	8.2	96.2	98.4	1731.6±126.4	1771.2 ±145.2	92.2 ±4.1	75.0±4.6

**Table 2:** Summary of stability of MCT under various storage conditions (n = 3).

Concentration (ng/mL)	Auto sampler ambient			Ambient 12 h			-20°C 30 d			Freeze-thaw		
	Found (ng/mL)	Accuracy (%)	RSD (%)	Found (ng/mL)	Accuracy (%)	RSD (%)	Found (ng/mL)	Accuracy (%)	RSD (%)	Found (ng/mL)	Accuracy (%)	RSD (%)
3	2.9± 0.1	96.6	3.5	3.1 ±0.2	102.9	7.6	2.8±0.4	93.6	13.8	2.7 ±0.3	88.9	13.0
180	183.4±9.7	101.9	5.3	190.8±9.2	106.0	4.8	177.1±20.4	98.4	11.5	162.2 ±8.3	90.1	5.1
1800	1879.2± 54.5	104.4	2.9	1657.8±89.5	92.1	5.4	1936.8 ±108.5	107.6	5.6	1918.8±188.0	106.6	9.8

**Table 3:** The main pharmacokinetic parameters of MCT after sublingual intravenous and intragastric administration (n=6).

Parameters	Unit	iv (3 mg/kg)	po (15 mg/kg)
AUC_(0-t)_	ng/mL*∗*h	2991.9 ±789.1	13215.0±5384.2
AUC_(0-*∞*)_	ng/mL*∗*h	3225.9 ±941.9	13259.7±5403.8
MRT_(0-t)_	H	2.6 ±1.2	1.6 ±0.7
MRT_(0-*∞*)_	h	4.5 ±3.3	1.6 ±0.7
t_1/2z_	h	7.1 ±3.7	2.6 ±1.5
t_max_	h	—	0.5±0.0
CL_z/F_	L/h/kg	1.0 ±0.4	1.3 ±0.5
V_z/F_	L/kg	9.3 ±3.6	4.5±3.2
C_max_	ng/mL	2553.8 ±340.2	9886.5 ±771.3

## Data Availability

The data used to support the findings of this study are included within the article.
